# ﻿Description of three new species of *Callyntrura* (Japonphysa) (Collembola, Entomobryidae) from China with the aid of DNA barcoding

**DOI:** 10.3897/zookeys.1187.109608

**Published:** 2023-12-21

**Authors:** Mei-Dong Jing, Yin-Huan Ding, Yi-Tong Ма

**Affiliations:** 1 School of Life Sciences, Nantong University, Nantong, Jiangsu 226000, China Nantong University Nantong China; 2 Department of Agronomy and Horticulture, Jiangsu Vocational College of Agriculture and Forestry, Jurong, Jiangsu 212400, China Jiangsu Vocational College of Agriculture and Forestry Jurong China

**Keywords:** Chaetotaxy, DNA sequence, identification key, Salininae, subgenus, taxonomy, Yoshii

## Abstract

*Callyntrura(s.l.)* Börner, 1906 is the largest genus of the subfamily Salininae and contains 11 subgenera and 98 species from all over the world (mainly Asia), with eight species recorded from China. In the present paper, three new species of *Callyntrura(s.l.)* are described from China: C. (Japonphysa) xinjianensis**sp. nov.**; C. (J.) tongguensis**sp. nov.** and C. (J.) raoi**sp. nov.** Their differences in colour pattern, chaetotaxy and other characters are slight, however distances of COI mtDNA support their validation as three new distinct species. A key to the Chinese *Callyntrura(s.l.)* is provided.

## ﻿Introduction

The genus *Callyntrura (s.l.)* Börner, 1906 was previously considered a member of the family Paronellidae, but now belongs to the family Entomobryidae (Godeiro et al. 2022). It is mainly characterized by the smooth dens, fusiform scales on body, the presence of frontal spines on the head and more than three teeth on the mucro. *Callyntrura* was subdivided into 11 subgenera on the base of labral chaetae, antennae, dental spines and other characters (Yoshii 1992). The subgenus Japonphysa was established by Yoshii in 1982 and it contains four species. The subgenus can be separated from the other subgenera of *Callyntrura* by the absence of modified labral chaetae and the presence of a blunt basal chaeta on the maxillary outer lobe.

*Callyntrura(s.l.)* specimens are medium-sized and their colour pattern plays a key role in its classification. So far, 97 species of *Callyntrura* have been described from Southeast and South Asia and one species from Africa and the descriptions of most species were quite simple (Bellinger et al. 1996–2023). Prior to this study, eight species belonging to four subgenera were described or reported from China (Ma 2013). Here we describe three new species of Callyntrura (Japonphysa) from China, based on their morphology and molecular data. A key to Chinese *Callyntrura(s.l.)* is also provided.

## ﻿Materials and methods

### ﻿Taxon sampling and specimen examinations

Specimens were collected with an aspirator and stored in 99% alcohol. They were mounted on glass slides in Marc André II solution, and were studied with a Leica DM2500 phase contrast microscope. Photographs were taken under a Leica DFC300 FX digital camera which mounted on the microscope and enhanced with Photoshop CS2 (Adobe Inc.). SEM photographs were taken under a ZEISS Gemini SEM 300 after the specimens were coated with a Leica EM ACE600. Type specimens are deposited in School of Life Sciences, Nantong University, Jiangsu, China.

The nomenclature of the dorsal macrochaetotaxy of the head and interocular chaetae are described following Jordana and Baquero (2005) and Mari-Mutt (1986). Labial chaetae are designated following Gisin (1964); labral and tergal chaetae of the body follow Szeptycki (1973, 1979); and teeth of the mucro follow Mitra (1974).

### ﻿Molecular analysis

DNA was extracted from one specimen per species by using an Ezup Column Animal Genomic DNA Purification Kit (Sangon Biotech, Shanghai, China) following the manufacturer’s standard protocols. Amplification of a 658 bp fragment of the mitochondrial COI gene was carried out using a Prime Thermal Cycler (TECHNE, Bibby Scientific Limited, Stone, Staffordshire, UK) in 25 μl volumes using Premix Taq polymerase system (Takara Bio, Otsu, Shiga, Japan). The primers and PCR progams followed Greenslade et al. (2011). All PCR products were checked on a 1% agarose gel electrophoresis. Successful products were purified and sequenced by Majorbio (Shanghai, China) on an ABI 3730XL DNA Analyser (Applied Biosystem, Foster City, CA, USA).

DNA sequences were assembled using Sequencher 4.5 (Gene Codes Corp), and then deposited in GenBank. Sequences were aligned by ClustalW implemented in MEGA 6 (Tamura et al. 2011) with default settings. Pairwise genetic distances were analyzed in MEGA 6 employing the Kimura 2-parameter (K2-P) model (Kimura 1980).

### ﻿Abbreviations

**Ant.** antennal segment(s);

**Th.** thoracic segment(s);

**Abd.** abdominal segment(s);

**mac** macrochaeta(e);

**mes** mesochaeta(e);

**ms** specialised microchaeta(e);

**sens** specialized ordinary chaeta(e).

## ﻿Results

**Figure 1.**
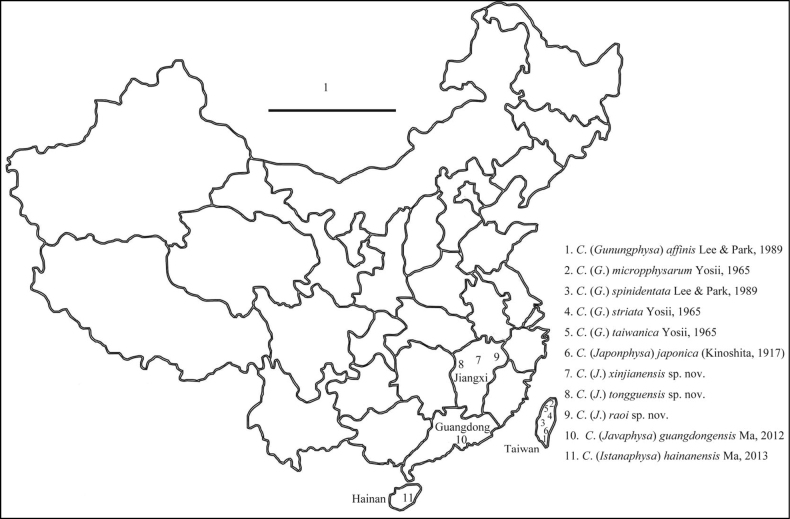
Record locality of all Chinese species of *Callyntrura(s.l.)* in China. Scale bar: 1000 km.

The distribution in China of the species described in present paper is shown in Fig. 1.

**Class Collembola Lubbock, 1873**

**Order Entomobryomorpha Börner, 1913**

**Family Entomobryidae Tömösvary, 1882**

### ﻿Genus Callyntrura

Taxon classificationAnimaliaEntomobryomorphaParonellidae

(s.l.) Börner, 1906

83B986E9-B9E8-5F35-9D01-06CA64C79E01

#### Diagnosis.

Moderate size, usually 2–3 mm; antennae four segmented and without apical bulb; eyes 8+8; frontal spines on head 4+4; scales present on body; dens smooth; mucro almost square and with more than three teeth.

### ﻿ Callyntrura (Japonphysa) xinjianensis sp. nov.

Taxon classificationAnimaliaEntomobryomorphaParonellidae

9A7435CB-DBB2-53B7-85DE-39A3D407854D

https://zoobank.org/3984F42A-FBF7-4331-86D1-F2EE71F16DAE

Figs 2–6 , 7–19 , 20–25 , 26–31 , 32–35 , 36–38 , 39–48 , Table 1

#### Type material.

***Holotype*.** ♀ on slide, China, Jiangxi Province, Nanchang City, Xinjian District, Meiling Town, Jiuxi Village, 12-XI-2020, 28°47'56"N, 115°45'11"E, 168 m asl, sample number 1243. ***Paratypes*.** 3♀♀ on slides, same data as holotype; 6♀♀ on slides, China, Jiangxi Province, Nanchang City, Xinjian District, Meiling Town, Shizifeng Park, 12-XI-2020, 28°49'26"N, 115°43'06"E, 100 m asl, sample number 1242. All collected by Y-T Ma.

#### Description.

***Size*.** Body length up to 2.75 mm.

**Figures 2–6.**
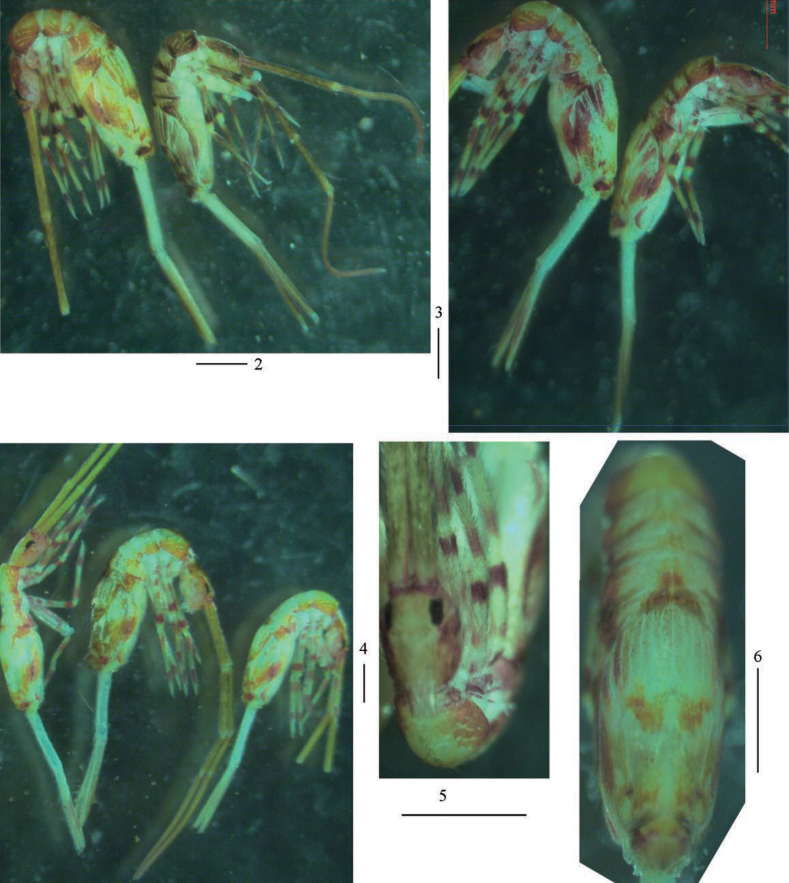
Habitus of *Callyntruraxinjianensis* sp. nov. **2–4** lateral view **5** dorsal view of head **6** dorsal view of trunk. Scale bars: 500 μm.

***Coloration*.** Ground colour pale yellow; eye patches dark blue; brown stripe present from head to Abd. III laterally; middle part of Abd. II–III with brown pigment; medial and posterior margin of Abd. IV with pair of irregular brown patches, respectively; brown pigment scattered on basal Ant. I and distal Ant. IV, legs, anterior part of ventral tube and distal dentes (Figs 2–6).

**Figures 7–19.**
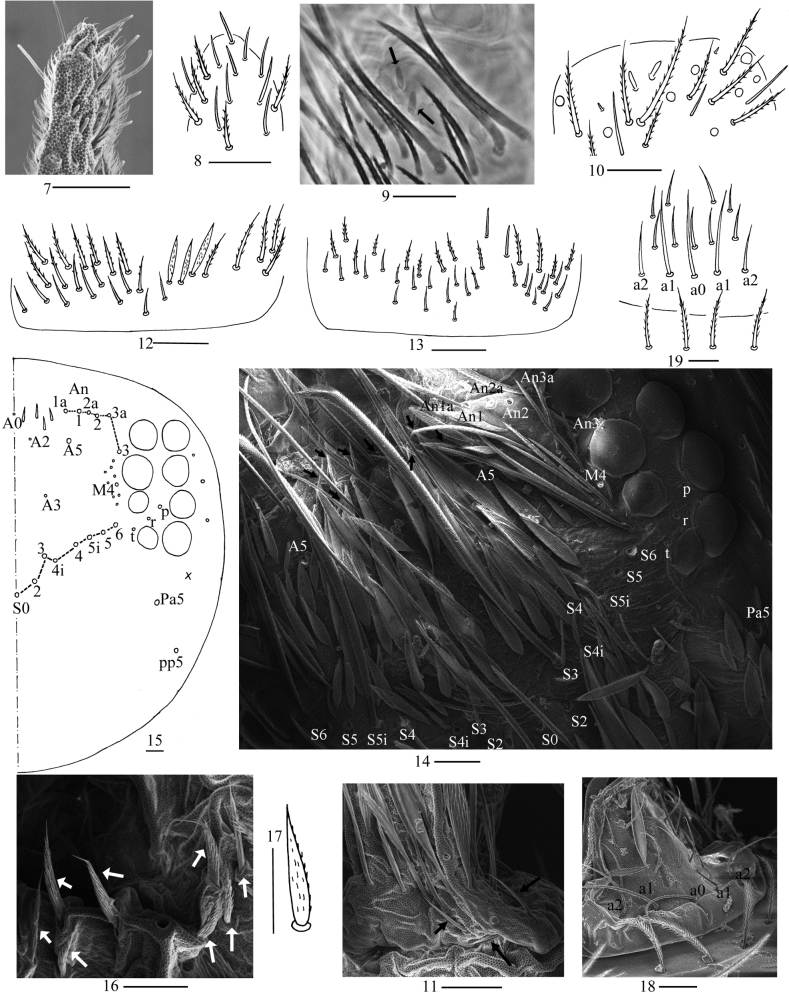
*Callyntruraxinjianensis* sp. nov. **7** SEM photomicrograph of apex of Ant. IV **8** apex of Ant. IV (dorsal view) **9** photomicrograph of distal Ant. III (arrow showing rod-like chaeta of Ant. III organ, ventral view) **10** distal Ant. III (ventral view) **11** SEM photomicrograph of basal Ant. I **12** basal Ant. I (dorsal view) **13** basal Ant. I (ventral view) **14** SEM photomicrograph of anterior part of dorsal head **15** dorsal head (right side) **16** SEM photomicrograph of frontal spines of head **17** frontal spine **18** SEM photomicrograph of prelabrum and labrum **19** prelabrum and labrum. Scale bars: 20 μm.

**Figures 20–25.**
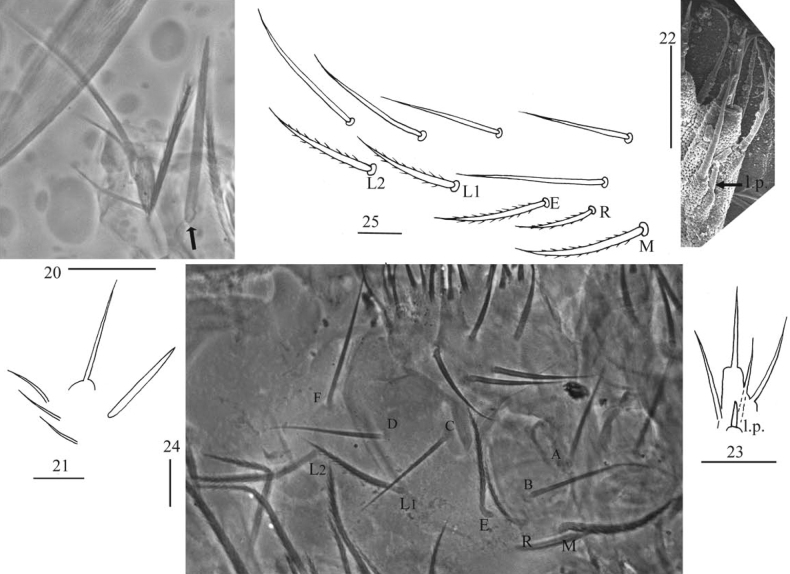
*Callyntruraxinjianensis* sp. nov. **20** photomicrograph of maxillary palp and outer lobe (arrow showing basal chaeta, right side) **21** maxillary palp and outer lobe (right side) **22** SEM photomicrograph of labial palp E (right side) **23** labial palp E (right side) **24** photomicrograph of labial base (left side) **25** labial base (left side). Scale bars: 20 μm.

***Head*.** Antenna not annulated and 1.37–1.57 times length of body. Ratio of Ant. I–IV as 1.00/0.85–1.00/0.49–0.72/1.37–2.00. Distal part of Ant. IV with many sensory chaetae and normal ciliate chaetae, without apical bulb (Figs 7, 8). Ant. III organ with two rod-like chaetae (Figs 9, 10). Basal Ant. I with smooth chaetae (Figs 11–13). Dorsal chaetotaxy of head as in Figs 14, 15, An series with six mac, A with four mac or mes, S with eight mac, P with two mac. Eyes 8+8, G & H smaller than others; interocular chaetae as p, r, t; frontal spines 4+4, all serrate (Figs 16, 17). Prelabral chaetae four, ciliate; labral chaetae 5, 5, 4, smooth and pointed, a0, a1 longer than a2; labral papillae absent (Figs 18, 19). Basal chaeta on maxillary outer lobe thick and blunt; sublobal plate with three smooth chaetae-like processes (Figs 20, 21). Lateral process (l. p.) of labial palp E differentiated, as thick as normal chaeta, with tip not reaching apex of papilla E (Figs 22, 23). Labial base with MREL_1_L_2_, all ciliate and R 0.51–0.77 length of M (Figs 24, 25).

**Figures 26–31.**
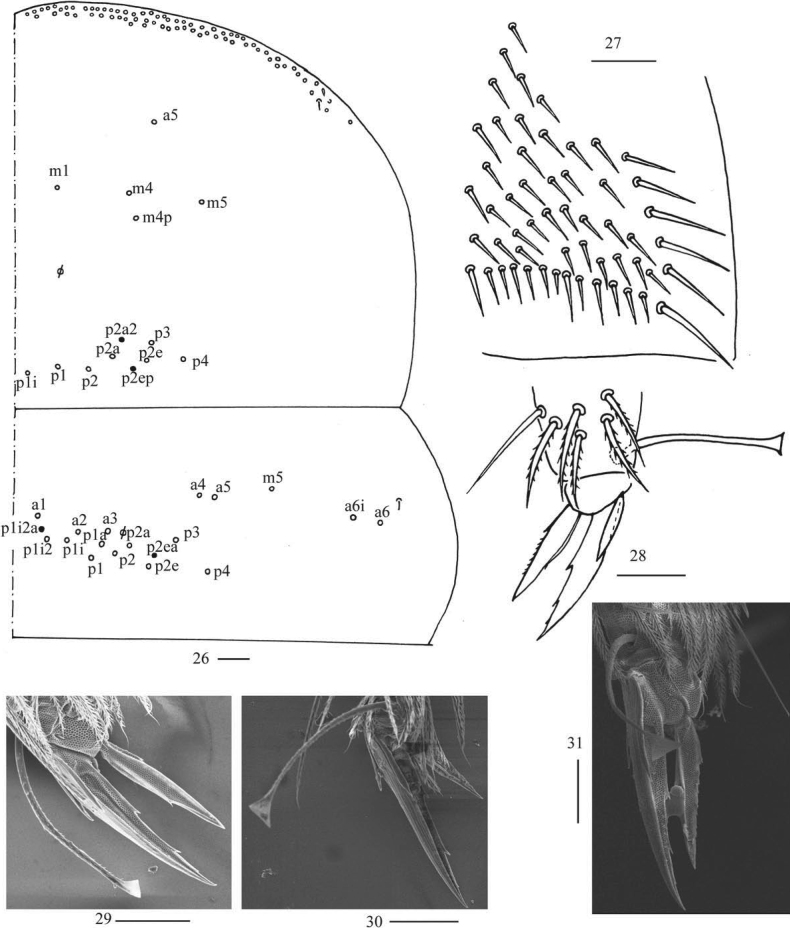
*Callyntruraxinjianensis* sp. nov. **26** chaetotaxy of Th. II–III (right side, solid black dot meaning absence) **27** trochanteral organ **28** hind foot complex (lateral view) **29** SEM photomicrograph of fore foot complex (lateral view) **30** SEM photomicrograph of middle foot complex (lateral view) **31** SEM photomicrograph of hind foot complex (lateral view). Scale bars: 20 μm.

**Figures 32–35.**
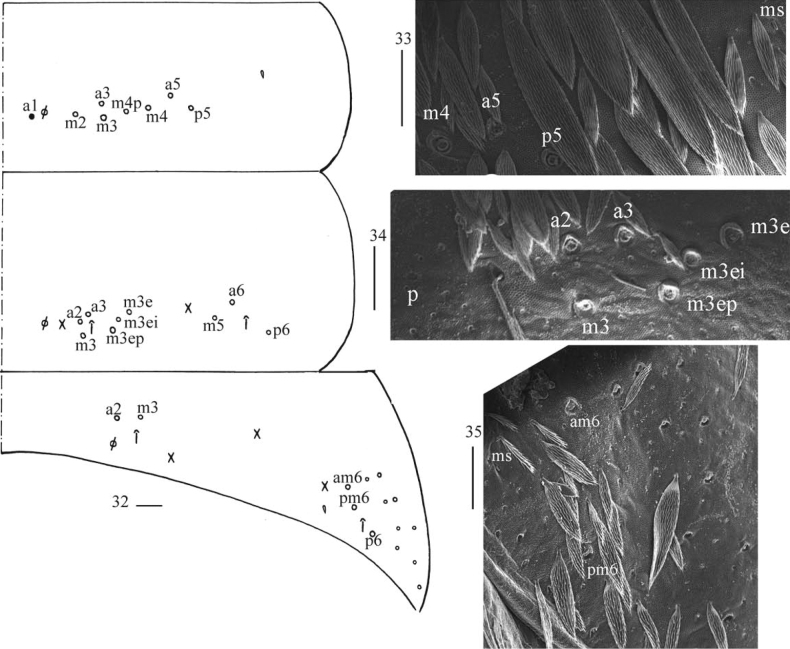
*Callyntruraxinjianensis* sp. nov. **32** cheatotaxy of Abd. I–III (right side, solid black dot meaning absence) **33** SEM photomicrograph of lateral Abd. I (right side) **34** SEM photomicrograph of central Abd. II (right side) **35** SEM photomicrograph of lateral Abd. III (right side). Scale bar: 20 μm.

**Figures 36–38.**
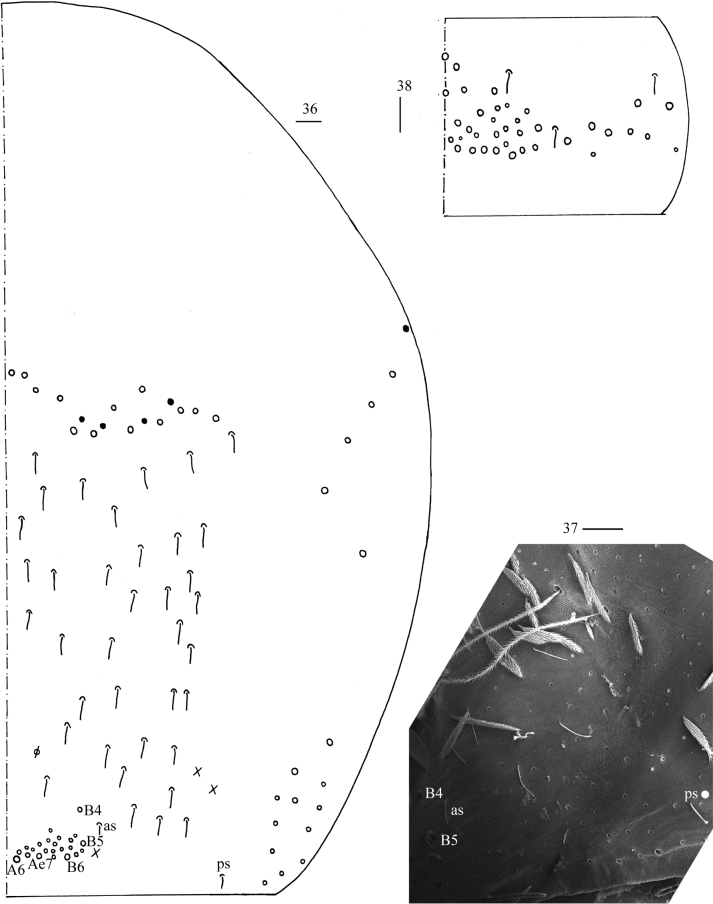
*Callyntruraxinjianensis* sp. nov. **36** chaetotaxy of Abd. IV (right side, solid black dot meaning absence) **37** photomicrograph of posterior-lateral Abd. IV (right side) **38** chaetotaxy of Abd. V (right side). Scale bars: 20 μm.

***Thorax*.** Tergal ms formula on Th. II–Abd. V as 1, 0/1, 0, 1, 0, 0, sens as 1, 1/0, 2, 2, 31–44, 3 (Figs 26, 32, 36, 38). Th. II with medial five (m1, m4, m4p, a5, m5) mac, usually posterior nine (p1i, p1, p2, p2a, p2e, p3, p4 always present, p2a2 or p2ep rarely absent) mac, one ms and one sens. Th. III with anterior-lateral five (a4, a5, m5, a6i, a6), usually posterior 12–14 (p1i2a and p2ea sometimes absent) mac, one sens (Fig. 26). Trochanteral organ with 55–64 chaetae (Fig. 27). Tenent hair clavate and slightly ciliate, 1.00–1.20 as long as inner edge of unguis; unguis with four inner teeth, most distal tooth very faint, basal pair located at 0.30–0.42 distance from base of inner edge of unguis, unpaired teeth at 0.65–0.70 and 0.82–0.90 distance from base, respectively; unguiculus lanceolate, with one median inner tooth and outer edge slightly serrate (Figs 28–31).

**Table 1.** Variation of tergal chaetotaxy of the new species (?, chaetotaxy not seen clearly).

Species	Specimen number	Th. II	Th. III	Abd. I	Abd. III	Abd. IV	
		posterior	posterior		lateral	medial	posterior
*Callyntruraxinjianensis* sp. nov.	1242-2A	9+?	12+13	7+7	?+?	16+17	23+24
	1242-2B	8+8	14+14	7+7	12+12	16+17	23+24
	1242-2C	8+8	12+13	7+7	13+13	16+17	17+17
	1242-3A	9+9	12+13	7+7	12+14	17+?	18+21
	1242-3B	9+9	13+13	7+7	11+13	15+16	17+17
	1242-3C	9+9	14+14	8+8	11+11	16+16	23+24
	1243-3A	9+9	13+13	7+7	12+?	15+?	14+18
	1243-3B	9+?	14+14	7+8	10+12	16+16	16+19
	1243-3C	9+9	12+13	8+8	14+?	?+?	18+23
	1243-3D	8+9	13+14	7+7	11+?	15+16	13+14
*C.tongguensis* sp. nov.	1235-3A	10+10	15+15	11+11	16+16	15+15	19+22
	1235-3B	10+10	14+15	11+11	14+14	17+17	20+23
	1235-3C	10+10	15+15	10+11	16+?	17+18	18+19
*C.raoi* sp. nov.	1244-1A	10+10	14+14	8+9	10+?	17+17	10+11
	1244-1B	10+10	14+14	?+?	15+15	15+15	14+18
	1244-1C	10+10	14+14	9+?	14+18	18+18	19+27
	1244-3A	9+9	14+14	9+9	14+?	15+15	22+24
	1244-3B	10+10	14+14	8+8	12+13	15+15	17+19
	1244-3C	10+10	14+14	8+8	12+?	13+14	10+11
	1244-4A	9+10	14+14	9+9	13+?	12+16	15+16
	1244-4C	9+9	14+14	9+9	17+?	13+14	12+12
	1244-4D	10+10	14+15	9+9	15+?	15+15	14+16

**Figures 39–48.**
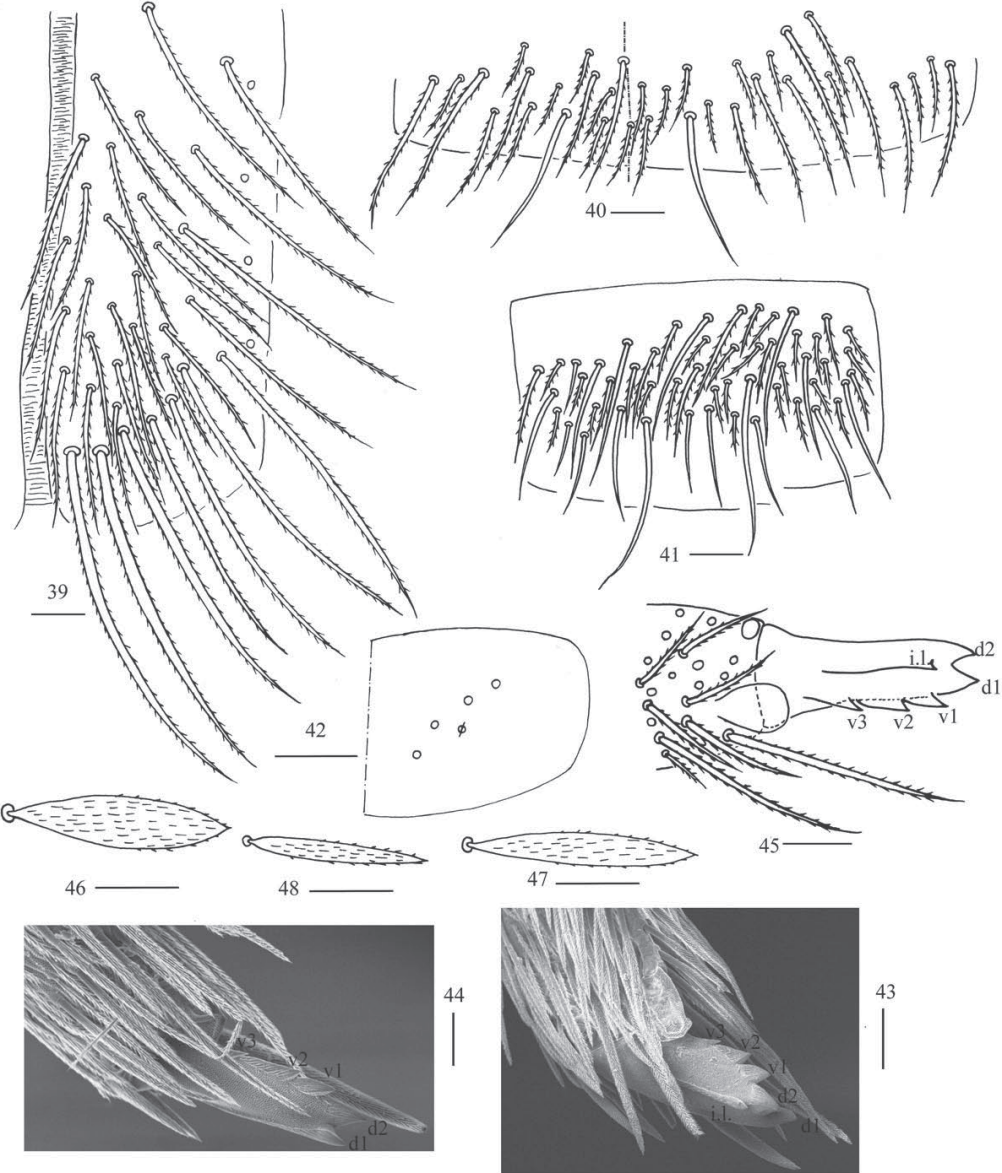
*Callyntruraxinjianensis* sp. nov. **39** anterior face of ventral tube **40** posterior face of ventral tube apically **41** lateral flap of ventral tube **42** manubrial plaque (dorsal view) **43** SEM photomicrographs of mucro (lateral view from internal side) **44** SEM photomicrographs of mucro (lateral view from external side) **45** mucro (upper view) **46, 47** scales on body **48** scale on antenna and furcula. Scale bars: 20 μm.

***Abdomen*.** Range of Abd. IV length as 6.71–13.75 times as dorsal axial length of Abd. III. Abd. I usually with seven (a3, a5, m2, m3, m4, m4i, p5, a1 rarely present) mac and one ms (Figs 32, 33). Abd. II with central six (a2, a3, m3, m3e, m3ei, m3ep), lateral three (m5, a6, p6) mac and two sens (Figs 32, 34). Abd. III with central two (a2, m3), lateral three (am6, pm6, p6) mac and 7–11 mes, one ms and two sens (Figs 32, 35). Abd. IV with 29–42 elongate and two (as, ps) normal sens, medial 15–17 and posterior 13–24 mac or mes, lateral 8–9 mac (Figs 36, 37). Abd. V with three sens (Fig. 38). Ventral tube with 18–22 (rarely 37) ciliate chaetae on each side anteriorly (Fig. 39); numerous ciliate chaetae and two apical smooth chaetae posteriorly (Fig. 40); 14–26 smooth and 5–36 ciliate chaetae on each lateral flap (Fig. 41). Manubrial plaque with four ciliate mac and one pseudopore (Fig. 42). Dens without spines. Mucro with six (v1, v2, v3, d1, d2, i.l.) teeth (Figs 43–45).

***Scales*.** Scales present on head, body, legs (Figs 46, 47); Ant. I–III and ventral side of manubrium and dens with narrower scales (Fig. 48). Ant. IV, ventral tube and tenaculum without scales.

#### Etymology.

Named after its locality: Xinjian District.

#### Ecology.

Found in the leaf litter, mainly composed of bamboo.

### ﻿ Callyntrura (Japonphysa) tongguensis sp. nov.

Taxon classificationAnimaliaEntomobryomorphaParonellidae

74784451-9396-5BB8-ADB7-39EB554B29AB

https://zoobank.org/E6007F8F-1468-44D3-B5F1-E209ED68279A

Figs 49–50 , 51–56 , 57–59 , 60 , 61, 62 , 63–70 , Table 1

#### Type material.

***Holotype*.** ♀ on slide, China, Jiangxi Province, Yichun City, Tonggu County, Tonggu Park, 9-XI-2020, 31°54'50"N, 114°22'36"E, 239 m asl, sample number 1235. ***Paratypes*.** 2 ♀♀ on slides, same data as holotype. All collected by Y-T Ma.

#### Description.

***Size*.** Body length up to 2.23 mm.

**Figures 49, 50.**
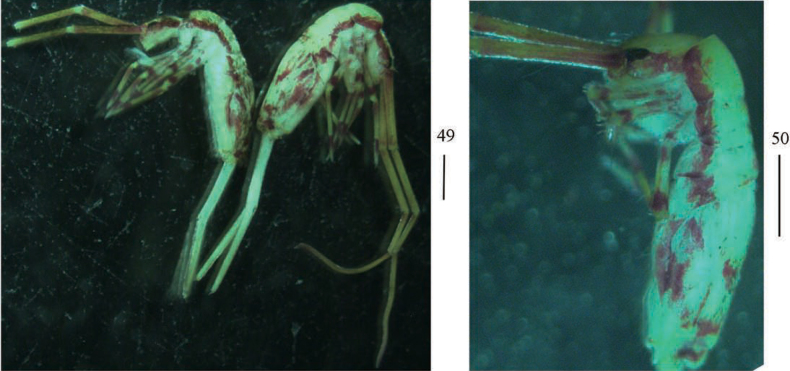
Habitus of *Callyntruratongguensis* sp. nov. (lateral view). Scale bars: 50 μm.

***Coloration*.** Ground colour pale yellow; antennae with scattered brown pigment; eye patches dark blue; brown stripe present from head to Abd. III laterally; middle part of Abd. II–III with brown pigment; medial and posterior margin of Abd. IV with pair of irregular brown patches, respectively; brown pigment scattered on antennae and legs (Figs 49, 50).

**Figures 51–56.**
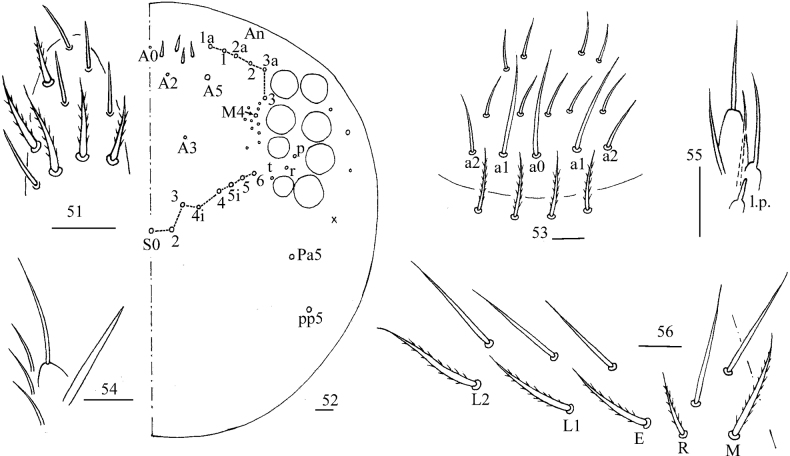
*Callyntruratongguensis* sp. nov. **51** apex of Ant. IV (dorsal view) **52** dorsal head (right side) **53** prelabrum and labrum **54** maxillary palp and outer lobe (right side) **55** labial palp E (right side) **56** labial base (left side). Scale bars: 20 μm.

***Head*.** Antenna not annulated and 1.45–1.49 times length of body. Ratio of Ant. I–IV as 1.00/0.90–0.98/0.58–0.63/1.50–1.54. Distal part of Ant. IV with many sensory chaetae and normal ciliate chaetae, without apical bulb (Fig. 51). Ant. III organ not clearly seen. Dorsal chaetotaxy of head as in Fig. 52, An series with six mac, A with four mac or mes, S with eight mac, P with two mac. Eyes 8+8, G & H smaller than others; interocular chaetae as p, r, t; frontal spines 4+4, all serrate. Prelabral chaetae four, ciliate; labral chaetae 5, 5, 4, smooth and pointed, a0, a1 longer than a2; labral papillae absent (Fig. 53). Basal chaeta on maxillary outer lobe thick and blunt; sublobal plate with three smooth chaetae-like processes (Fig. 54). Lateral process (l. p.) of labial palp E differentiated, as thick as normal chaeta, with tip not reaching apex of papilla E (Fig. 55). Labial base with MREL_1_L_2_, all ciliate and R 0.53–0.68 length of M (Fig. 56).

**Figures 57–59.**
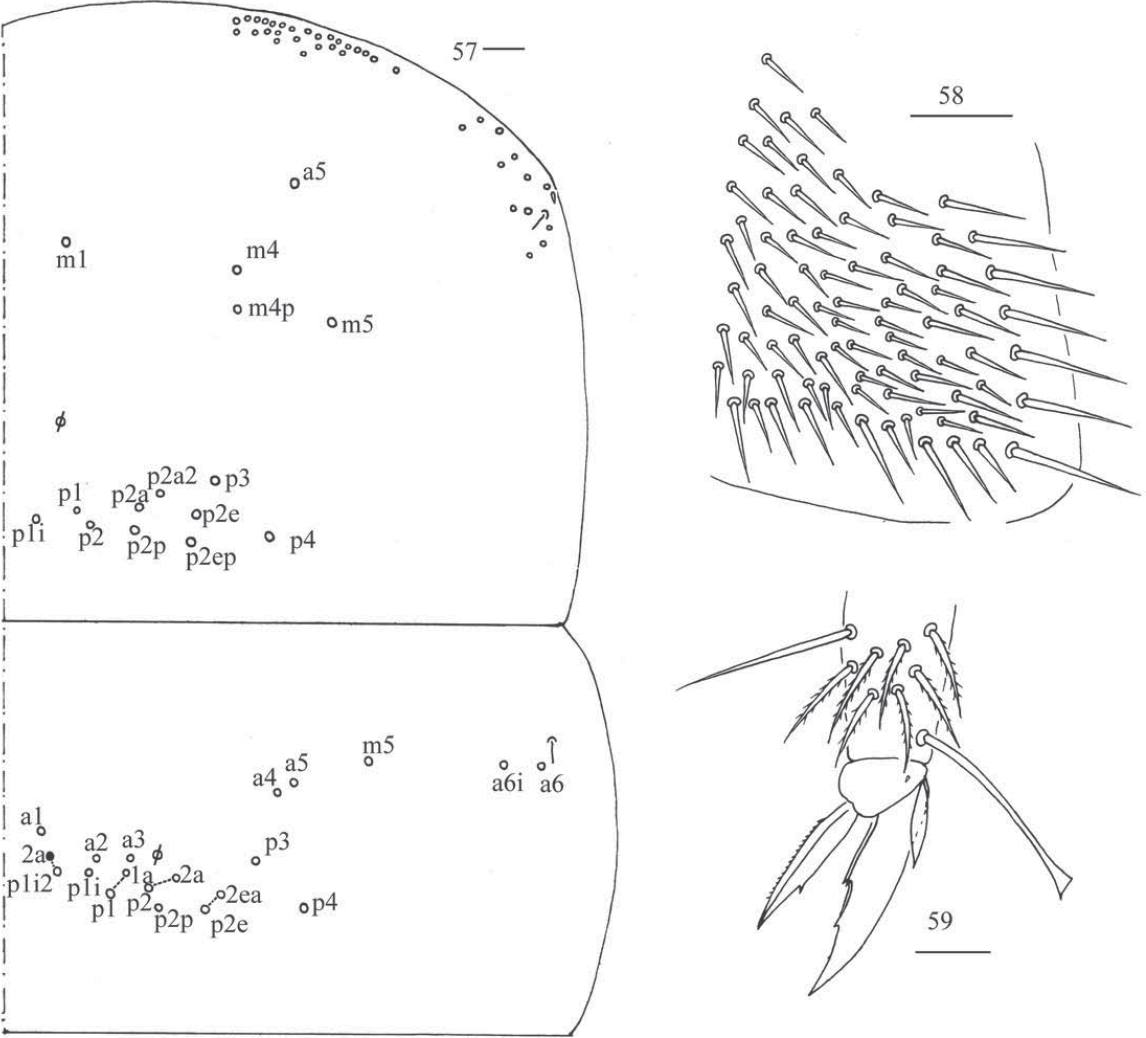
*Callyntruratongguensis* sp. nov. **57** chaetotaxy of Th. II–III (right side, solid black dot meaning absence) **58** trochanteral organ **59** hind foot complex (lateral view). Scale bars: 20 μm.

**Figure 60.**
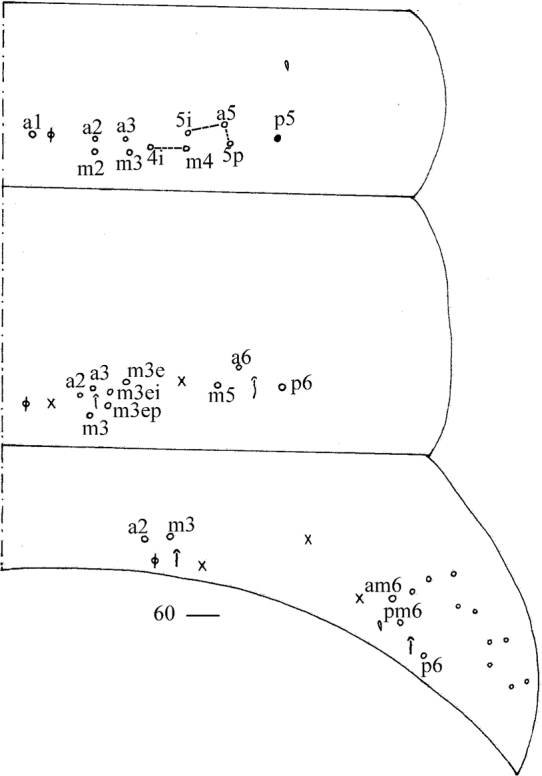
Chaetotaxy of Abd. I–III of *Callyntruratongguensis* sp. nov. (right side, solid black dot meaning absence). Scale bar: 20 μm.

**Figures 61, 62.**
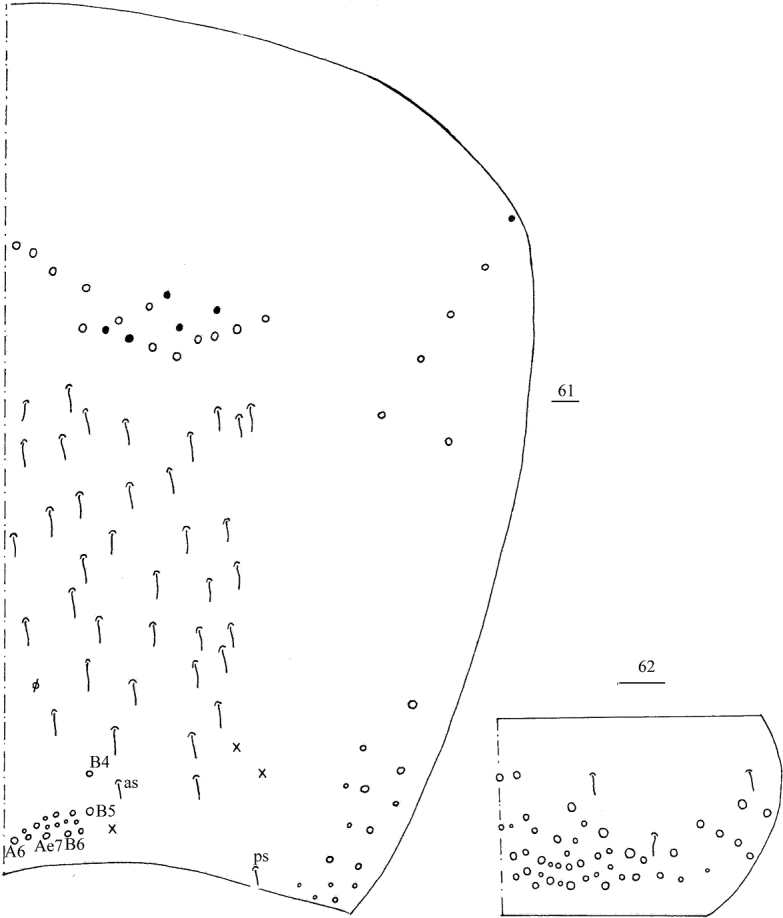
*Callyntruratongguensis* sp. nov. **61** chaetotaxy of Abd. IV (right side, solid black dot meaning absence) **62** chaetotaxy of Abd. V (right side). Scale bars: 20 μm.

***Thorax*.** Tergal ms formula on Th. II–Abd. V as 1, 0/1, 0, 1, 0, 0, sens as 1, 1/0, 2, 2, 37–41, 3 (Figs 57, 60–62). Th. II with medial five (m1, m4, m4p, a5, m5) mac, posterior ten (p1i, p1, p2, p2a, p2a2, p2p, p2e, p2ep, p3, p4) mac, one ms and one sens. Th. III with anterior-lateral five (a4, a5, m5, a6i, a6), usually posterior 15 (p1i2a rarely absent) mac, one sens (Fig. 57). Trochanteral organ with 63–81 chaetae (Fig. 58). Tenent hair clavate, 1.10–1.15 as long as inner edge of unguis; unguis with four inner teeth, most distal tooth very faint, basal pair located at 0.37–0.41 distance from base of inner edge of unguis, unpaired teeth at 0.58–0.61 and 0.70–0.78 distance from base, respectively; unguiculus lanceolate, with one inner tooth and outer edge slightly serrate (Fig. 59).

**Figures 63–70.**
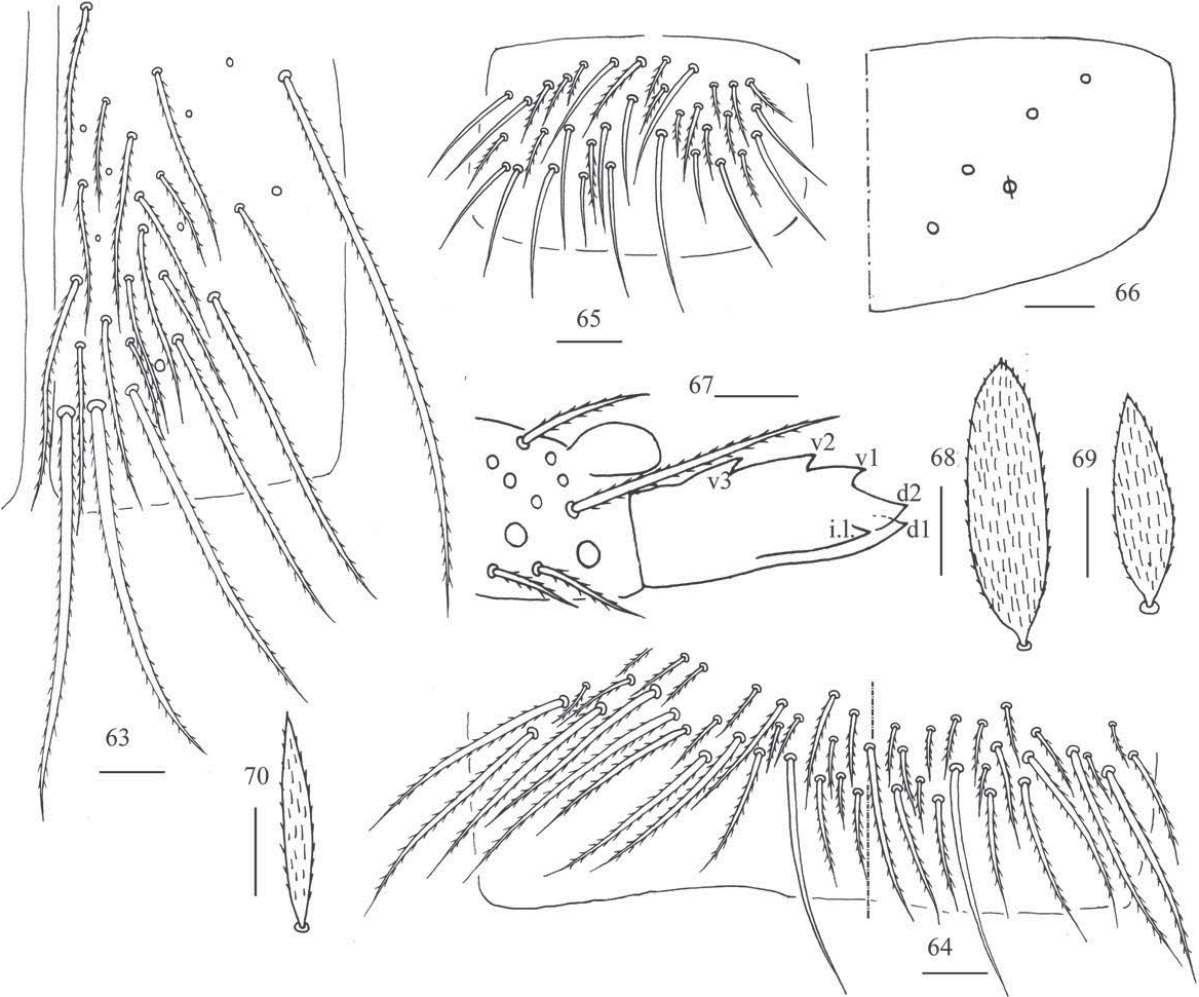
*Callyntruratongguensis* sp. nov. **63** anterior face of ventral tube **64** posterior face of ventral tube apically **65** lateral flap of ventral tube **66** manubrial plaque (dorsal view) **67** mucro (lateral view from internal side) **68, 69** scales on body **70** scale on antenna and furcula. Scale bars: 20 μm.

***Abdomen*.** Range of Abd. IV length as 10.00–12.00 times as dorsal axial length of Abd. III. Abd. I usually with 11 (a1–3, a5i, a5, a5p, m2–4, m4i, p5 rarely absent) mac and one ms. Abd. II with central six (a2, a3, m3, m3e, m3ei, m3ep), lateral three (m5, a6, p6) mac. Abd. III with central two (a2, m3), lateral three (am6, pm6, p6) mac and 10–14 mes (Fig. 60). Abd. IV with 35–39 elongate and two (as, ps) normal sens, medial 15–18 and posterior 18–23 mac or mes, lateral 8–9 mac (Fig. 61). Abd. V with three sens (Fig. 62). Ventral tube with 25–29 ciliate chaetae on each side anteriorly (Fig. 63); numerous ciliate chaetae and 2–3 apical smooth chaetae posteriorly (Fig. 64); 17–24 smooth and 10–17 ciliate chaetae on each lateral flap (Fig. 65). Manubrial plaque with four ciliate mac and one pseudopore (Fig. 66). Dens without spines. Mucro with six (v1, v2, v3, d1, d2, i.l.) teeth (Fig. 67).

***Scales*.** Scales present on head, body, legs (Figs 68, 69); Ant. I–III and ventral side of manubrium and dens with narrower scales (Fig. 70). Ant. IV, ventral tube and tenaculum without scales.

#### Etymology.

Named after its locality: Tonggu County.

#### Ecology.

Found in the leaf litter.

### ﻿ Callyntrura (Japonphysa) raoi sp. nov.

Taxon classificationAnimaliaEntomobryomorphaParonellidae

6DC3FB5B-662F-52A8-ADFF-E507B7BFDEA6

https://zoobank.org/D2240BC8-FC64-4525-B39B-8AC1E7C68077

Figs 71–73 , 74–80 , 81–83 , 84 , 85, 86 , 87–95 , Table 1

#### Type material.

***Holotype*.** 1♀ on slide, China, Jiangxi Province, Shangrao City, Dexing City, Raoshoukun Memorial Park, 13-XI-2020, 28°57'20"N, 117°34'08"E, 88 m asl, sample number 1244. ***Paratypes*.** 8 ♀♀ on slides, same data as holotype. All collected by Y-T Ma.

#### Description.

***Size*.** Body length up to 2.45 mm.

**Figures 71–73.**
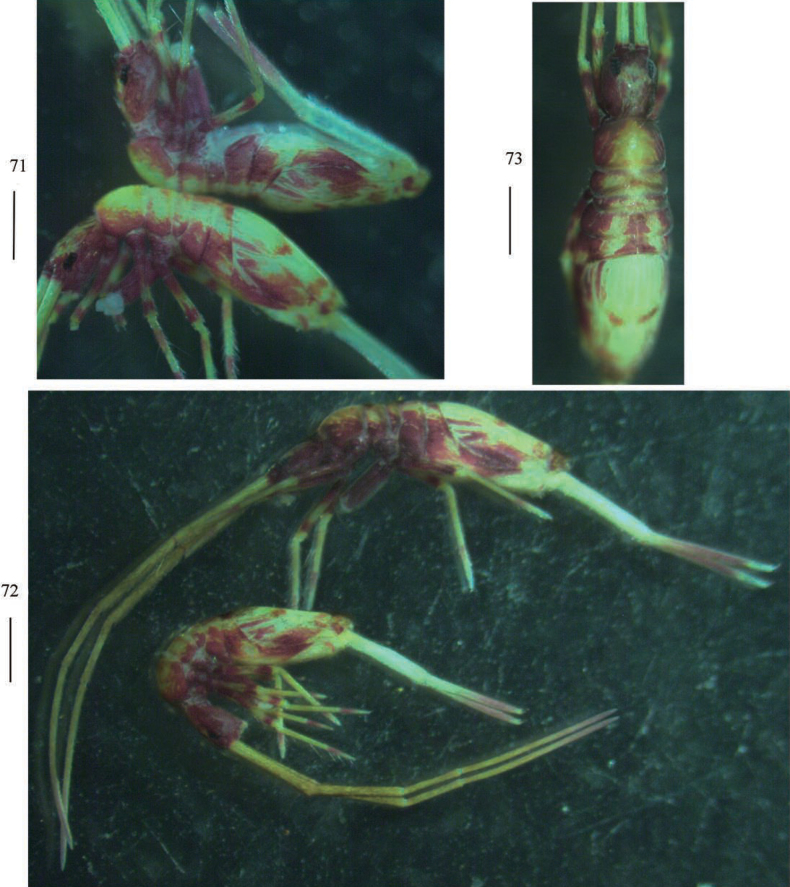
Habitus of *Callyntruraraoi* sp. nov. **71, 72** lateral view **73** dorsal view. Scale bars: 500 μm.

***Coloration*.** Ground colour pale yellow; eye patches dark blue; head almost brown entirely; brown stripe present from Th. II to Abd. III laterally; ventral tube, middle part of Abd. II–III brown pigmented; medial and posterior margin of Abd. IV with pair of irregular brown patches, respectively; brown pigment present also on legs, distal Ant. IV and distal dentes (Figs 71–73).

**Figures 74–80.**
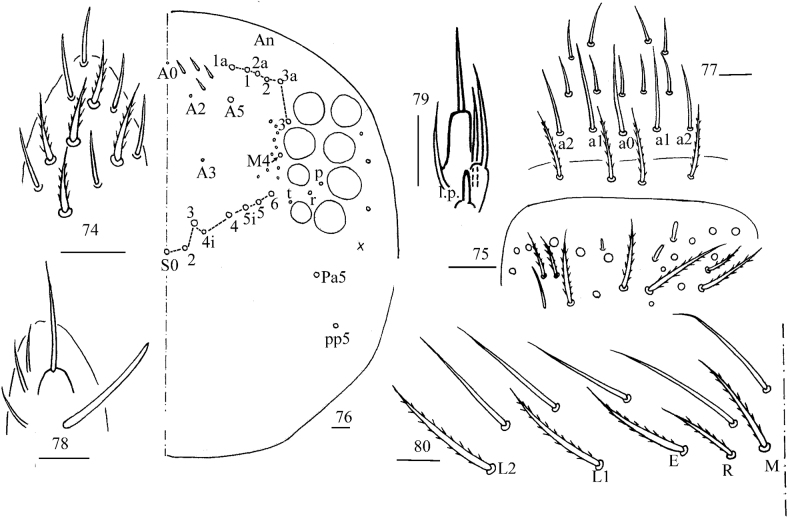
*Callyntruraraoi* sp. nov. **74** apex of Ant. IV (dorsal view) **75** distal Ant. III (ventral view) **76** dorsal head **77** prelabrum and labrum **78** maxillary palp and outer lobe (right side) **79** labial palp E (right side) **80** labial base (left side). Scale bars: 20 μm.

***Head*.** Antenna not annulated and 1.32–1.53 times length of body. Ratio of Ant. I–IV as 1.00/0.92–1.00/0.60–0.75/1.60–2.04. Distal part of Ant. IV with many sensory chaetae and normal ciliate chaetae, without apical bulb (Fig. 74). Ant III organ with two rod-like chaetae (Fig. 75). Dorsal chaetotaxy of head as in Fig. 76, An series with six mac, A with four mac or mes, S with eight mac, P with two mac. Eyes 8+8, G & H smaller than others; interocular chaetae as p, r, t; frontal spines 4+4, all serrate. Prelabral chaetae four, ciliate; labral chaetae 5, 5, 4, smooth and pointed, a0, a1 longer than a2; labral papillae absent (Fig. 77). Basal chaeta on maxillary outer lobe thick and blunt; sublobal plate with three smooth chaetae-like processes (Fig. 78). Lateral process (l. p.) of labial palp E differentiated, as thick as normal chaeta, with tip not reaching apex of papilla E (Fig. 79). Labial base with MREL_1_L_2_, all ciliate and R 0.51–0.77 length of M (Fig. 80).

**Figures 81–83.**
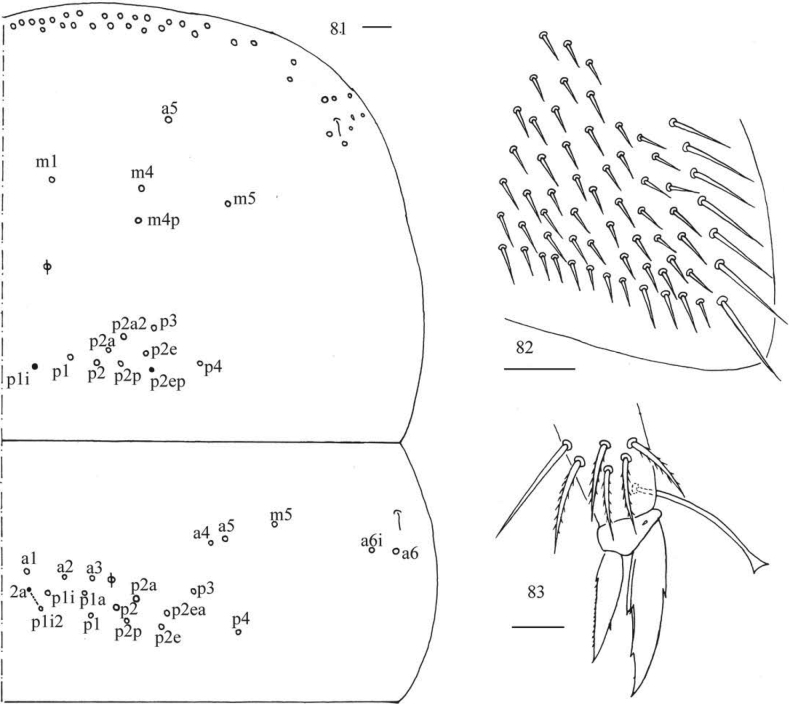
*Callyntruraraoi* sp. nov. **81** chaetotaxy of Th. II–III (right side, solid black dot meaning absence) **82** trochanteral organ **83** hind foot complex (lateral view). Scale bars: 20 μm.

**Figure 84.**
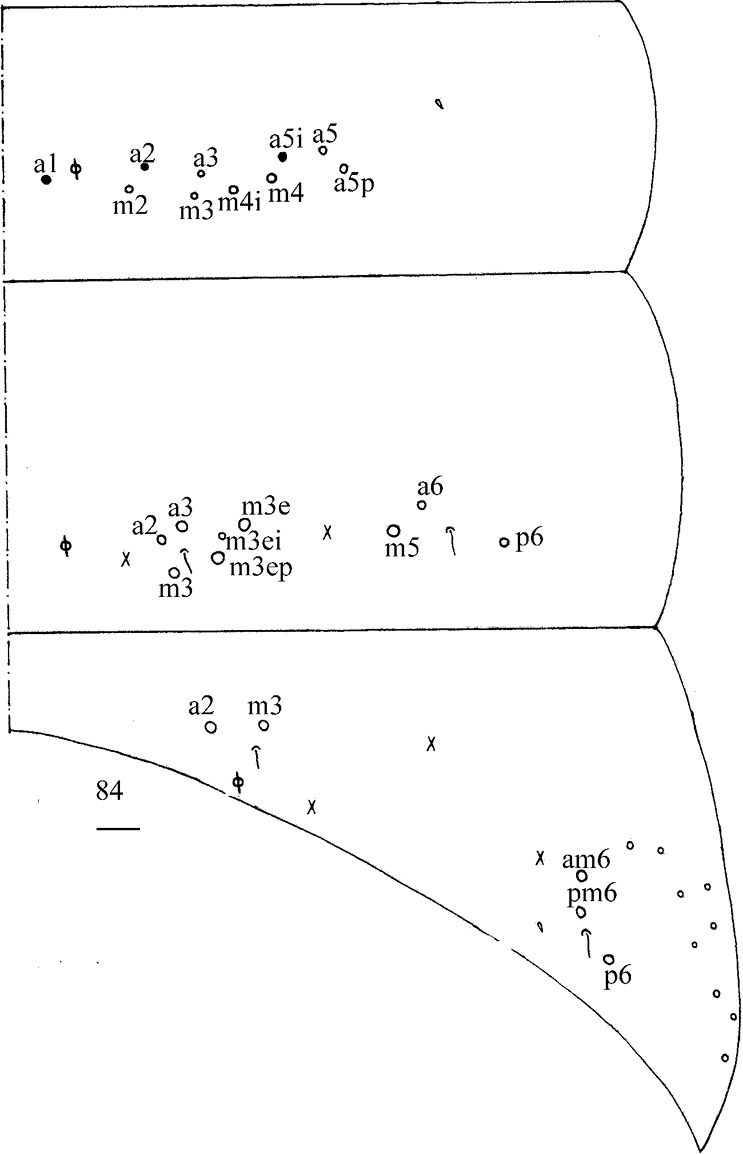
Chaetotaxy of Abd. I–III of *Callyntruraraoi* sp. nov. (right side, solid black dot meaning absence). Scale bar: 20 μm.

**Figures 85, 86.**
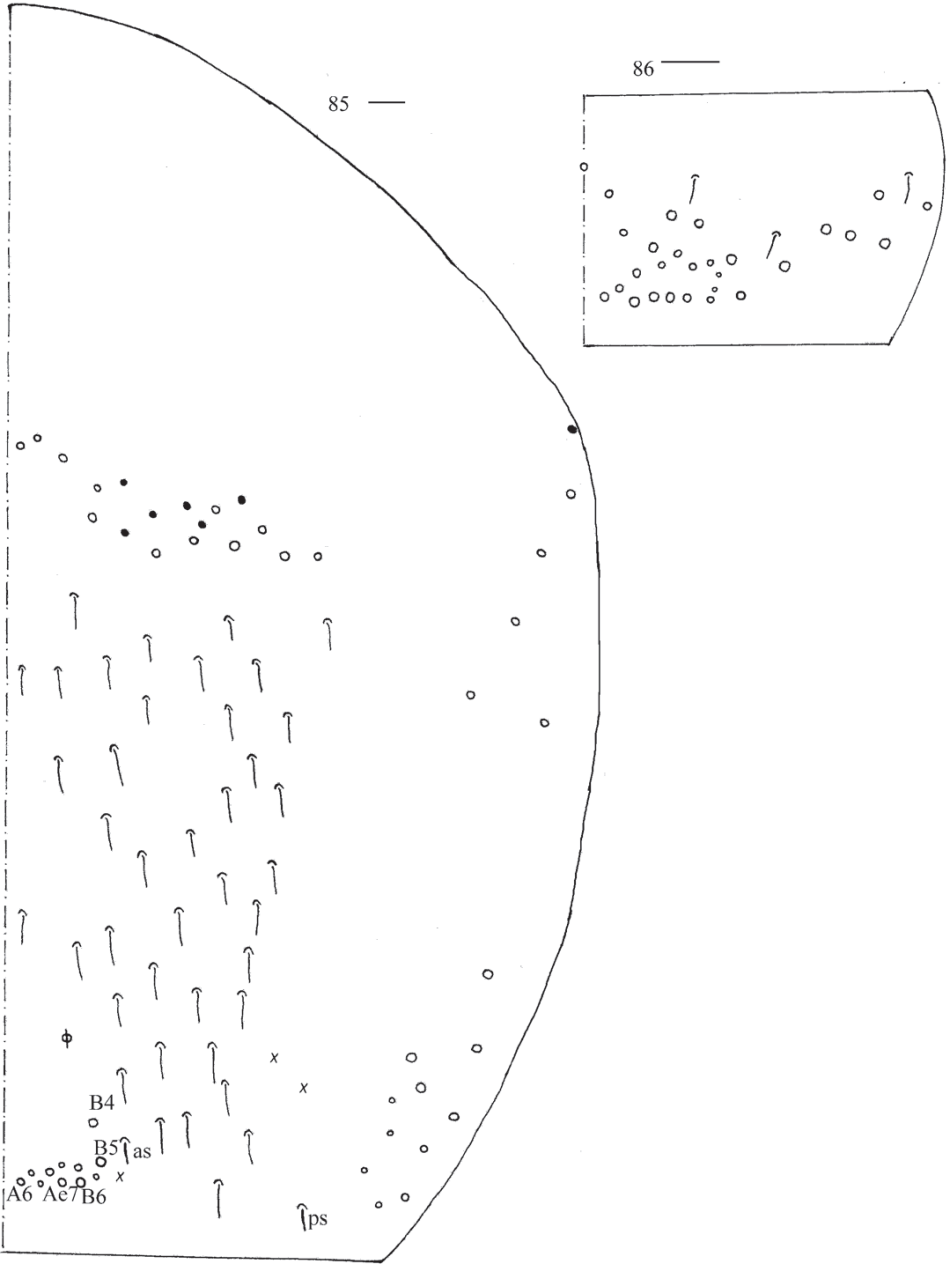
*Callyntruraraoi* sp. nov. **85** chaetotaxy of Abd. IV (right side, solid black dot meaning absence) **86** chaetotaxy of Abd. V (right side). Scale bars: 20 μm.

***Thorax*.** Tergal ms formula on Th. II–Abd. V as 1, 0/1, 0, 1, 0, 0, sens as 1, 1/0, 2, 2, 29–42, 3 (Figs 81, 84–86). Th. II with medial five mac, usually posterior 10 (p1i, p1, p2, p2a, p2a2, p2p, p2e, p2ep, p3, p4, p1i or p2ep rarely absent) mac, one ms and one sens. Th. III with anterior-lateral five (a4, a5, m5, a6i, a6), usually posterior 14 mac (p2a rarely present), one sens (Fig. 81). Trochanteral organ with 63–64 chaetae (Fig. 82). Tenent hair clavate, 1.06–1.20 as long as inner edge of unguis; unguis with four inner teeth, most distal tooth very faint, basal pair located at 0.31–0.42 distance from base of inner edge of unguis, unpaired teeth at 0.66–0.68 and 0.82–0.89 distance from base, respectively; unguiculus lanceolate, with one inner tooth and outer edge slightly serrate (Fig. 83).

**Figures 87–95.**
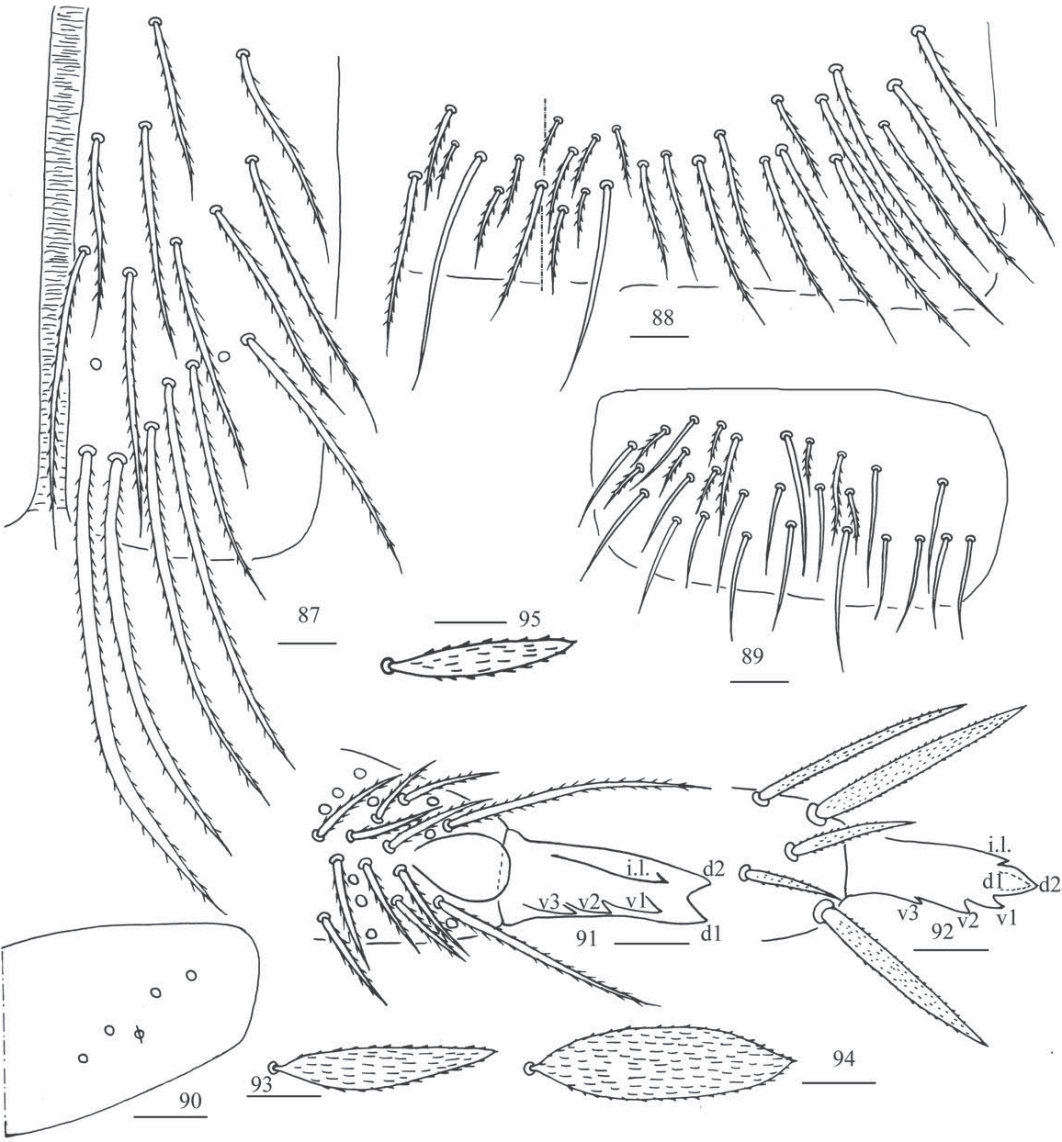
*Callyntruraraoi* sp. nov. **87** anterior face of ventral tube **88** posterior face of ventral tube apically (partially) **89** lateral flap of ventral tube **90** manubrial plaque (dorsal view) **91** mucro (upper view) **92** mucro (ventral view) **93, 94** scales on body **95** scale on antenna and furcula. Scale bars: 20 μm.

***Abdomen*.** Range of Abd. IV length as 7.02–10.67 times as dorsal axial length of Abd. III. Abd. I with 8–9 (a3, a5, a5p, m2–4, m4 always present, a1, a2 or a5i sometimes absent) mac and one ms. Abd. II with central six (a2, a3, m3, m3e, m3ei, m3ep), lateral three (m5, a6, p6) mac. Abd. III with central two (a2, m3), lateral three (am6, pm6, p6) mac and 8–13 mes (Fig. 84). Abd. IV with 27–40 elongate and two (as, ps) normal sens, medial 14–18 and posterior 10–27 mac or mes, lateral 8–9 mac (Fig. 85). Abd. V with three sens (Fig. 86). Ventral tube with 17 ciliate chaetae on each side anteriorly (Fig. 87); numerous ciliate chaetae and two apical smooth chaetae posteriorly (Fig. 88); 14–21 smooth and 6–19 ciliate chaetae on lateral flap (Fig. 89). Manubrial plaque with four ciliate mac and one pseudopore (Fig. 90). Dens without spines. Mucro with six (v1, v2, v3, d1, d2, i.l.) teeth (Figs 91, 92).

***Scales*.** Scales present on head, body, legs (Figs 93, 94); Ant. I–III and ventral side of manubrium and dens with narrower scales (Fig. 95). Ant. IV, ventral tube and tenaculum without scales.

#### Etymology.

“*raoi*” (in apposition) refers to Lieutenant General Shoukun Rao, who made immortal achievements in the Chinese People’s War of Resistance against Japanese Aggression and the War of Liberation.

#### Ecology.

Found in the leaf litter.

#### Remarks.

The three new species are very similar in overall chaetotaxy, colour pattern and other characters. The chaetotaxy of each studied specimen is listed in Table 1, and the differences between these three species are slight. One main difference in chaetotaxy is that Abd. I has 7(8), 11, 8–9 mac in C. (J.) xinjianensis sp. nov., C. (J.) tongguensis sp. nov. and C. (J.) raoi sp. nov., respectively. Another difference is p2p mac on Th. II is absent in C. (J.) xinjianensis sp. nov., but present in the latter two new species. One main difference in colour pattern between them is that only the lateral side of the head is brown pigmented in C. (J.) xinjianensis sp. nov., and C. (J.) tongguensis sp. nov., but almost the entire head is brown in C. (J.) raoi sp. nov. The brown pigment on the ventral tube is present anteriorly in C. (J.) xinjianensis sp. nov., absent in C. (J.) tongguensis sp. nov. and present almost entirely in C. (J.) raoi sp. nov.

The subgenus Japonphysa contains four species at present: C. (J.) japonica (Kinoshita, 1917), C. (J.) oligosetosa Kim, Rojanavongse & Lee, 1999, C. (J.) semilineata Yosii, 1961 and C. (J.) unilineata Yosii, 1961. The differences between the three new species and the four known species are great, especially in chaetotaxy of body (Table 2).

**Table 2.** Comparison between the new species and all known species of Callynthrura (Japonphysa).

Characters	C. (J.) xinjianensis sp. nov.	C. (J.) tongguensis sp. nov.	C. (J.) raoi sp. nov.	C. (J.) japonica	C. (J.) oligosetosa	C. (J.) semilineata	C. (J.) unilineata
Brown pigment on head	laterally	laterally	almost entirely	entirely	laterally	laterally	laterally
Brown pigment on ventral tube	anteriorly	absent	almost entirely	entirely	absent	absent	absent
Chaetae on labial base	MREL_1_L_2_	MREL_1_L_2_	MREL_1_L_2_	MRel_1_l_2_*	not known	not known	MRel_1_L_2_
Posterior mac on Th. II	8–9 (p2p absent)	10 (p2p present)	9–10 (p2p present)	7*	0	4	4
Posterior mac on Th. III	12–14	15	14	10*	0	9	7
Mac on Abd. I	7 (rarely 8)	11 (rarely 10)	8–9	6*	7	7	7
Central mac on Abd. II	6	6	6	5*	4	5	5
Central mac on Abd. III	2	2	2	2*	1	2	2
Inner teeth on unguis	4	4	4	3*	4	3	3–4
Anterior chaetae on ventral tube	18–22	25–29	17	not known	7	not known	not known

* based on Yoshii’s description (Yoshii 1982).

##### ﻿Molecular results

Sequenced individuals in the present study had a mean K2-P distance of COI sequences between 0.190–0.197 (about 19%). The shortest interspecific distance was 0.190 between *C.tongguensis* sp. nov. and *C.raoi* sp. nov. and the longest was 0.197 between *C.tongguensis* sp. nov. and *C.xinjianensis* sp. nov. (Table 3). Therefore, the interspecific distances of COI between the three new species were more than the accepted barcoding gap recently reported for the species of Entomobryidae (Zhang et al. 2018b) and Tomoceridae (Yu et al. 2018). The molecular distances coincided with the morphological divergences, thus further supporting the distinction of the three species.

**Table 3.** Genetic distances (mean K2-P divergence) of the COI sequences between the new described species.

Species	C. (J.) tongguensis sp. nov.	C. (J.) raoi sp. nov.	C. (J.) xinjianensis sp. nov.	GenBank Accession Numbers
C. (J.) tongguensis sp. nov.				OQ940723
C. (J.) raoi sp. nov.	0.190			OQ940724
C. (J.) xinjianensis sp. nov.	0.197	0.196		OQ940725

## ﻿Discussion

Colour pattern usually plays a very important role in the classification of Collembola and many species were described based on it previously. Although colour pattern is a good character and intraspecific variability is low in *Callyntrura* taxa, it is sometimes very difficult for taxonomists to distinguish those different species who share similar colouration. DNA barcoding is a good tool to separate species with a similar colour pattern and well used in classification in some genera of Collembola, such as *Coecobrya* (Nilsai et al. 2017; Zhang et al. 2018a), *Dicranocentrus* (Zhang et al. 2018b) and *Tomocerus* (Yu et al. 2016, 2017; Gong et al. 2018).

### ﻿Key to the Chinese species of *Callyntrura(s.l.)*

**Table:** 

1	No labral chaetae modified (subgenus Japonphysa)	**2**
–	Part or all chaetae on the first row of labrum modified	**5**
2	Pigment present on Th. II–Abd. III entirely	**C. (Japonphysa) japonica (Kinoshita, 1917)**
–	Pigment mainly present on Th. II–Abd. III laterally	**3**
3	Pigment present on head entirely, Abd. I with 8–9 mac	**C. (J.) raoi sp. nov.**
–	Pigment present on head only laterally, Abd. I usually with 7 or 11 mac	**4**
4	Abd. I usually with 7 mac, p2p mac absent	**C. (J.) xinjianensis sp. nov.**
–	Abd. I usually with 11 mac, p2p mac present	**C. (J.) tongguensis sp. nov.**
5	Three median chaetae on the first row of labrum modified	**6**
–	All chaetae on the first row of labrum modified (subgenus Gunungphysa)	**7**
6	Abd. III with 1 dorsal mac	**C. (Javaphysa) guangdongensis Ma, 2012**
–	Abd. III with 2 dorsal mac	**C. (Istanaphysa) hainanensis Ma, 2013**
7	Dens with spines	**C. (Gunungphysa) spinidentata Lee & Park, 1989**
–	Dens without spines	**8**
8	Abd. I with 3 mac	**C. (G.) taiwanica Yosii, 1965**
–	Abd. I with more than 3 mac	**9**
9	Abd. I with 5 mac	**10**
–	Abd. I with 9 mac	**C. (G.) affinis Lee & Park, 1989**
10	A longitudinal stripe present from Th. II to Abd. III laterally	**C. (G.) striata Yosii, 1965**
–	Pigment diffused on body	**C. (G.) microphysarum Yosii, 1965**

## Supplementary Material

XML Treatment for Callyntrura

XML Treatment for Callyntrura (Japonphysa) xinjianensis

XML Treatment for Callyntrura (Japonphysa) tongguensis

XML Treatment for Callyntrura (Japonphysa) raoi

## ﻿Additional information

### Author ORCIDs

Mei-Dong Jing http://orcid.org/0000-0002-8498-9498

Yin-Huan Ding https://orcid.org/0000-0002-8002-7056

Yi-Tong Ма http://orcid.org/0000-0002-8660-0503

### Data availability

All of the data that support the findings of this study are available in the main text.
